# Thermodynamic and Technological Compatibility of Polyvinyl Chloride, Thermoplastic Polyurethane, and Bio-Plasticizer Blends

**DOI:** 10.3390/polym17091149

**Published:** 2025-04-23

**Authors:** Yitbarek Firew Minale, Ivan Gajdoš, Pavol Štefčák, Ľudmila Dulebová, Tomasz Jachowicz, Tamás Szabó, Andrea Ádámné Major, Kálmán Marossy

**Affiliations:** 1 Institute of Energy, Ceramics and Polymer Technology, University of Miskolc, 3515 Miskolc-Egyetemváros, Hungary; minale.yitbarek@student.uni-miskolc.hu (Y.F.M.); tamas.szabo.mak@uni-miskolc.hu (T.S.); marossyk@gmail.com (K.M.); 2 Department of Chemical Engineering, Bahir Dar Institute of Technology, Bahir Dar University, Bahir Dar 6000, Ethiopia; 3 Department of Technologies, Materials and CAx, Faculty of Mechanical Engineering, Technical University of Košice, 04001 Košice, Slovakia; pavol.stefcak@tuke.sk (P.Š.); ludmila.dulebova@tuke.sk (Ľ.D.); 4 Department of Polymer Processing, Faculty of Mechanical Engineering, Lublin University of Technology, 20-618 Lublin, Poland; t.jachowicz@pollub.pl; 5 Department of Innovative Vehicles and Materials, Johnvon Neumann University, 6000 Kecskemét, Hungary; major.andrea@nje.hu

**Keywords:** polymer blends, polyvinyl chloride (PVC), thermoplastic polyurethane (TPU), bio-plasticizer, thermodynamic compatibility, technological compatibility

## Abstract

Polymer blending enhances material properties by combining different polymers, which requires careful consideration of both thermodynamic and technological compatibility. This study investigates the compatibility of polyvinyl chloride (PVC), thermoplastic polyurethane (TPU), and a bio-plasticizer in blends produced via roll milling at various mixing ratios. Compatibility and morphology were analyzed using thermally stimulated discharge (TSD), dynamic mechanical analysis (DMA), and scanning electron microscopy (SEM), while mechanical and thermal properties were assessed by mechanical testing and thermogravimetric analysis (TGA). The PVC/TPU (100/30) blend exhibited superior phase compatibility over PVC/TPU (100/50), as indicated by a single relaxation peak in TSD and DMA, along with a more homogeneous morphology and enhanced tensile properties. The PVC/TPU/bio-plasticizer (100/20/50) blend showed a well-balanced mechanical performance and improved phase homogeneity. The TSD peak maxima trends for the TPU/bio-plasticizer blend highlighted the bio-plasticizer’s dual role in enhancing flexibility at low concentrations while restricting molecular mobility at higher concentrations. TGA revealed TPU’s positive effect on PVC’s degradation profile, while the bio-plasticizer reduced thermal stability. These findings demonstrate that blending PVC, TPU, and bio-plasticizer creates compatible materials with enhanced and diverse properties, making them suitable for industrial applications.

## 1. Introduction

Polymer blending is a widely used technique for designing and producing new materials with enhanced and tailored properties by combining existing polymers [1,2]. In recent years, research and development in the field of polymers have increasingly focused on blending approaches to create new materials or modify existing ones, rather than synthesizing new monomers. This shift stems from the ability of polymer blends to effectively address technological challenges, enhance desired properties without significantly compromising others, and offer a diverse range of material properties through controlled composition adjustments. Furthermore, polymer blending is a time-efficient and cost-effective strategy that utilizes already established production processes and equipment, thereby minimizing the financial risks associated with the development of entirely new monomer systems [1,3,4,5].

Polyvinyl chloride (PVC) is one of the most widely used polymers globally, ranking as the third most produced thermoplastic [6,7]. Naturally white and brittle, PVC is valued for its high rigidity, flame retardancy, chemical resistance, and low cost, making it suitable for a wide range of applications in construction, transportation, and various industrial sectors [8,9,10,11]. However, its inherent limitations, including low elasticity and thermal stability, restrict its use in certain applications [12]. In contrast, thermoplastic polyurethane (TPU) is an elastomeric thermoplastic polymer known for its high elasticity, thermal stability, chemical resistance, and abrasion resistance [13,14,15]. TPU consists of block-structured linear polymer chains, characterized by alternating long, low-polarity segments (soft segments) and short, high-polarity segments (hard segments), which are covalently bonded to form block copolymers [16,17].

Glycerol diacetate monolaurate is a bio-plasticizer produced from waste cooking oils via transesterification and acetylation processes. Sourced from renewable and biodegradable materials, it mitigates the toxic effects and reduces dependence on petroleum-based plasticizers [18]. This additive enhances polymer processing and properties, particularly flexibility and toughness. When polymers are heated, thermal energy disrupts intermolecular forces, increasing molecular spacing and softening the material. The addition of a plasticizer facilitates this effect by embedding its molecules between polymer chains, preventing them from approaching each other, even after cooling. This process, known as plasticization, significantly alters the mechanical properties of polymers [19,20,21]. By blending PVC, TPU, and a bio-plasticizer, the good properties of each component are expected to reinforce one another, resulting in materials with enhanced properties.

The degree of compatibility among the components critically influences the final properties and performance of polymer blends [22]. A blend is considered thermodynamically incompatible if it exhibits distinct transition temperatures corresponding to its parent polymers, leading to phase separation, weak interfacial adhesion, and poor physical properties. Conversely, a polymer blend is thermodynamically compatible if it displays a single transition temperature, indicating molecular-level miscibility over a broad range of temperatures and compositions, which is associated with a negative free energy of mixing. This results in enhanced mechanical properties and improved phase cohesion [23,24].

Beyond thermodynamic considerations, technological compatibility is a practical concept that cannot be strictly defined through mathematical expressions. A polymer blend is considered technologically compatible when its components are miscible at a macroscale, appear homogeneous (i.e., without significant repulsive interactions), and demonstrate improved, useful, or application-relevant properties [25].

Conventional plasticizers, such as phthalates, have raised environmental and health concerns due to their potential toxicity [26]. Consequently, bio-based plasticizers derived from renewable sources have gained significant attention as sustainable alternatives. For instance, a study [27] synthesized an acetylated-fatty acid methyl ester-citric acid ester (AC-FAME-CAE) plasticizer from waste cooking oil and citric acid, demonstrating improved mechanical properties in PVC films, comparable to those of traditional petroleum-based plasticizers. While some other studies have also examined the compatibility and properties of PVC-based blends, including PVC/plasticizers, PVC/polyurethane (PU), and PVC/polyethylene (PE) systems using various techniques [28,29], there remains a gap in research comprehensively exploring systems combining PVC, TPU, and bio-plasticizer, addressing their compatibility and performance across various blend formulations. Moreover, with its renewable origin, glycerol diacetate monolaurate, sourced from abundant and underutilized waste streams (i.e., waste cooking oils) [30], offers a highly sustainable and cost-effective alternative to other bio-based plasticizers.

This study investigates the development of new polymeric materials by combining PVC, TPU, and a bio-based plasticizer in various blend formulations. A comprehensive analysis using TSD, DMA, SEM, TGA, and mechanical testing was conducted to evaluate the compatibility and performance of these blends, while FTIR was used to confirm the purity and composition of the bio-plasticizer. The findings provide valuable insights into the blends’ compatibility, performance, and potential industrial applications.

## 2. Materials and Methods

### 2.1. Materials

The suspension-type polyvinyl chloride (PVC) with a K-value of 70 and the polyester-based thermoplastic polyurethane (TPU) used in this study were supplied by BorsodChem Zrt, (Kazincbarcika, Hungary). Glycerol diacetate monolaurate, a low-molecular-weight bio-plasticizer, was obtained from Rikevita Fine Chemical & Food Industry Co., Ltd. (Shanghai, China). It has a molecular weight of 330.47 g/mol and a density of 0.99 g/cm^3^.

### 2.2. Methods

#### 2.2.1. Preparation of the Blends

PVC was initially pre-mixed with commercial-grade additives, including a CaZn stabilizer and E-Wax lubricant. The required amounts of TPU and PVC were then blended with the bio-plasticizer using a 10 L laboratory mixer (MTI Mischtechnik, Detmold, Germany). The formulations, with varying PVC, TPU, and bio-plasticizer ratios, as outlined in Table 1, were subsequently processed on an electrically heated roll mill (Schwabenthan 150 U, Berlin, Germany) at 175 °C. The roll milling was carried out at a constant speed ratio of 1:1 and a rotation speed of 21 rpm, facilitating efficient softening and mechanical shear mixing. The material was repeatedly cut and randomly repositioned between the rotating hot rolls to ensure effective mixing. The resulting roll-milled sheets, with thicknesses ranging from approximately 0.4 to 0.6 mm, were then compression molded at the same temperature to produce smooth, uniform sheets.

#### 2.2.2. Sample Preparation for TSD Test

**Figure 1:**
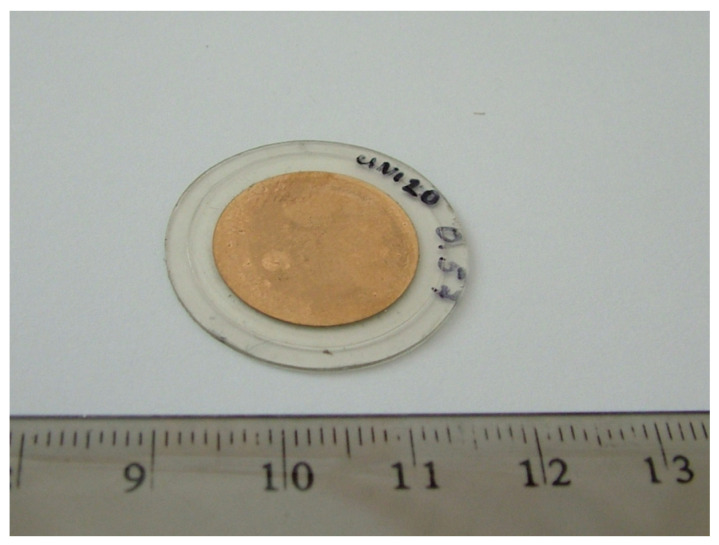
Gold-coated sample prepared for TSD testing.

Following the preparation of PVC, TPU, and their blend sheets, as described in Section 2.2.1, disk-shaped samples with a diameter of 26 mm and a thickness of approximately 0.5 mm were cut. To ensure conductive, gap-free surfaces and minimize noise from partial discharge during the TSD test, the samples were gold-coated using a sputter coater. This process was conducted under a low-pressure argon atmosphere (5–7 Pa), with an accelerating voltage of 200 V applied for gold sputtering. A thin gold layer (4–8 nm thick, 18 mm in diameter) was then deposited on the surface of the samples. The gold-coated sample is shown in Figure 1.

#### 2.2.3. Sample Preparation for Tensile Test

For tensile testing, polymer sheets and their blends, which were previously compression molded, were precisely cut into standardized dumbbell-shaped specimens using a die cutter (CTC-001162, ZUND, Altstätten, Switzerland). The dumbbell geometry, with dimensions including a gauge length of 33 mm, a gauge width of 6 mm, and a thickness of 0.6 mm, was selected to ensure uniform stress distribution during testing. This shape directs deformation to the narrow central region while minimizing premature failure at the specimen’s ends.

#### 2.2.4. TSD Test for Polymers and Their Blends

TSD measurements were conducted using a TSCII instrument (SETARAM, Caluire, France). The prepared samples were mounted on a specimen holder within the instrument’s sealed chamber. To eliminate impurities and enhance thermal conductivity, the chamber was evacuated and flushed with helium gas, which served as an efficient heat transfer medium. The samples were polarized at 120 °C, followed by controlled cooling to −120 °C at a rate of 5 °C/min, using liquid nitrogen as the cooling medium. The heating phase was conducted at the same rate to ensure precise depolarization current measurements. Each experiment lasted approximately two hours. Further details regarding the instrument, testing procedure, and evaluation method are well described in [31].

#### 2.2.5. TSD Test for the Bio-Plasticizer, Glycerol Diacetate Monolaurate

The procedure described in Section 2.2.4 was followed, with the main difference that gold plating was not required, as the fluid ensures gap-free contact with the electrode. A disk-shaped borosilicate glass filter paper (7 mm in diameter, 0.2 mm in thickness, and a nominal pore size of 2.6 µm) was used as a fluid carrier. To ensure uniform saturation, the glass fiber paper was thoroughly soaked with the bio-plasticizer before being placed in the specimen holder of the TSD instrument. Borosilicate glass filter paper was selected due to its high chemical resistance and low polarizability, minimizing interference with the TSD signal. The procedure of TSD tests on fluid substances is described in detail in [32,33].

#### 2.2.6. Fourier Transform Infrared (FTIR) Spectroscopy

FTIR analysis of the bio-plasticizer, glycerol diacetate monolaurate, was performed to verify its purity and composition. A Bruker Tensor 27 FTIR spectrometer (Bruker Optik GmbH, Ettlingen, Germany), equipped with a Gladi ATR accessory featuring a diamond crystal, was used. Spectra were recorded over the range of 400–4000 cm^−1^ with a resolution of 4 cm^−1^, averaging 128 scans per measurement. Data processing and spectral interpretation were conducted using OPUS software, version 7.5 (Bruker Optik GmbH), with characteristic absorption bands identified based on literature references to confirm the molecular structure and detect potential impurities.

#### 2.2.7. Dynamic Mechanical Analysis (DMA)

Dynamic mechanical analysis (DMA) was conducted using a Metravib DMA 25 (ACOEM Group, Limonest, France). Rectangular specimens (25 mm × 12 mm × 0.6 mm) were tested in tensile mode over a temperature range of −120 °C to 120 °C, with a controlled heating rate of 2 °C/min. Measurements were performed at a constant oscillatory frequency of 10 Hz, applying a static amplitude of 0.5 mm and a dynamic amplitude of 0.1 mm. Liquid nitrogen was used to regulate the temperature during low-temperature measurements.

#### 2.2.8. Scanning Electron Microscopy (SEM) Analysis

The morphology of the polymer blends was examined using a Hitachi SU5000 scanning electron microscope (Hitachi High-Technologies Corporation, Tokyo, Japan). To enhance phase contrast, samples were swelled in methyl ethyl ketone (MEK) for 24 h to selectively dissolve the more soluble phase, facilitating phase separation visualization. The specimens were then cross-sectioned and dried at 70 °C for 24 h to remove residual solvent and stabilize the structure. Dried samples were mounted on carbon stubs and sputter-coated with gold to ensure conductivity. SEM imaging was performed under high vacuum at an accelerating voltage of 15 kV and an emission current of 3.2 nA. High-resolution micrographs were captured using a secondary electron (SE) detector in ICE mode at 20,000× magnification, providing a detailed visualization of phase morphology and phase separation.

#### 2.2.9. Tensile Testing

Tensile testing of pure polymers and their blends was conducted using a computer-controlled tensile testing machine (Instron, Norwood, MA, USA) equipped with a 1 KN load cell. The tests were carried out at room temperature with a crosshead speed of 10 mm/min. Dumbbell-shaped specimens with standardized dimensions were securely mounted in the machine’s grips, with the lower grip fixed and the upper grip mobile. A uniaxial tensile load was progressively applied along the specimen’s longitudinal axis at a constant rate until failure. Throughout the test, the applied load (monitored via a load cell) and the corresponding elongation (tracked using an extensometer) were continuously recorded. Key parameters, including tensile strength, elongation at break, and Young’s modulus, were documented from the results.

#### 2.2.10. Hardness Measurement

The hardness of polymers and their blends was evaluated by measuring the penetration depth of an indenter into the test samples. Shore A hardness was determined using a 5–10 mm steel cone head under a 10 N applied force, whereas Shore D hardness was measured using a pin-shaped indenter with a 50 N applied force. The samples were placed on a hard, flat surface with a small gap between them and the indenter. During testing, the indenter was pressed down perpendicularly onto the sample for a duration of 3 s, and the readings were recorded. The final hardness value was obtained by averaging measurements taken from five different positions on the samples.

#### 2.2.11. Thermogravimetric Analysis (TGA)

Thermogravimetric analysis (TGA) was performed using a Q600 DSC-TGA instrument (TA Instruments, New Castle, DE, USA) to evaluate the thermal stability and degradation behavior of the polymer blends. Approximately 10.0 mg of each sample was placed in a platinum pan and heated from 30 °C to 600 °C at a constant rate of 10 °C/min under an argon atmosphere with a flow rate of 100 mL/min. Mass loss was recorded as a function of temperature to determine key thermal degradation parameters, including onset decomposition temperature and residual char content.

## 3. Results and Discussion

### 3.1. TSD Results of Polymer Blends

**Figure 2:**
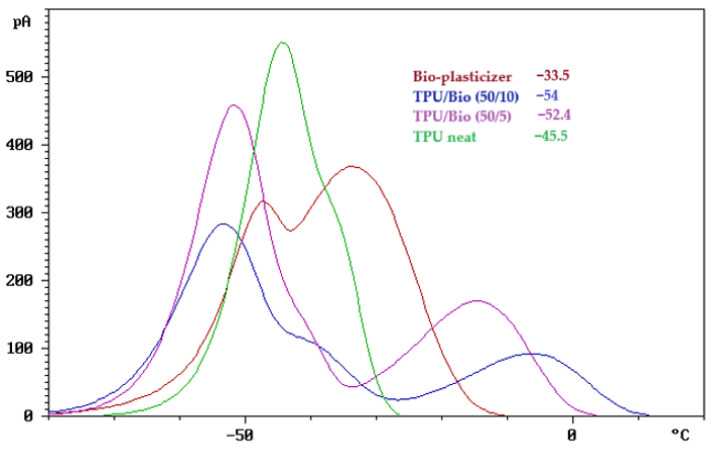
TSD curves of TPU/bio-plasticizer blends.

**Figure 3:**
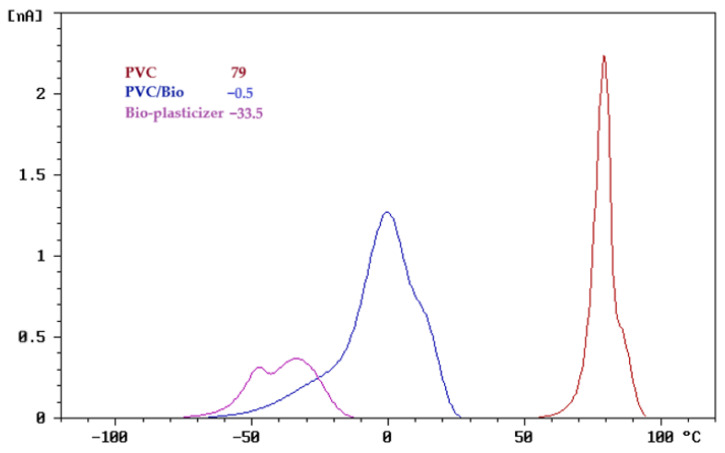
TSD curve of PVC/bio-plasticizer blend.

**Figure 4:**
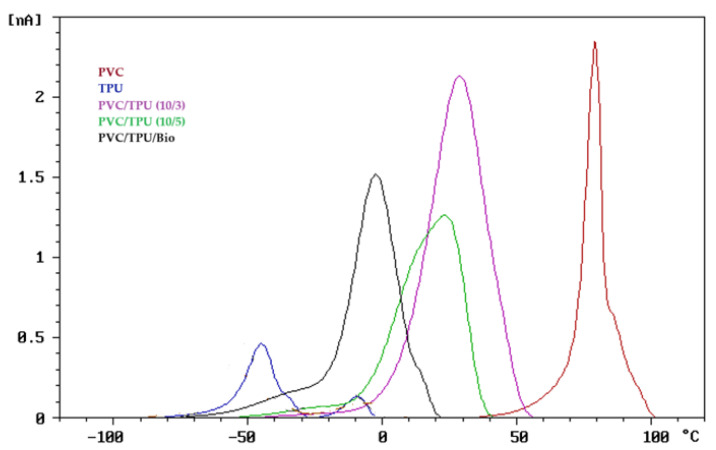
TSD curve of PVC/TPU/bio-plasticizer and PVC/TPU blends.

To assess the compatibility of the polymer blends, the thermally stimulated discharge (TSD) curves, normalized to an electric field of 1 kV/mm and an electrode area of 30 cm^2^, are presented in Figure 2, Figure 3 and Figure 4.

#### 3.1.1. TSD Curve Analysis of Polymer Blends

Figure 2 shows the TSD curves of neat TPU and TPU blended with a bio-based plasticizer, obtained at a heating rate of 5 °C/min. The current response (pA) is plotted as a function of temperature (°C), revealing distinct charge relaxation phenomena associated with the molecular mobility of different polymer segments.

The TSD response of neat TPU (green curve) exhibits two primary relaxation processes. The prominent peak at −45.5 °C corresponds to the depolarization of charges associated with the relaxation of the soft segments. A second, less pronounced peak appears at a slightly higher temperature, around −35 °C, which is attributed to the relaxation of the hard segments. This process is less detectable in the neat TPU curve, as it typically occurs at higher temperatures due to the rigid nature and limited mobility of these segments.

When the bio-plasticizer is added, it interacts with both the soft and hard segments of the TPU, resulting in the plasticization of each segment and the appearance of distinct peaks. The peaks observed in the blends at −54 °C (TPU/bio-plasticizer 50/10) and −52 °C (TPU/bio-plasticizer 50/5) are attributed to the plasticization of the soft segments. This interaction with the bio-plasticizer enhances chain mobility and flexibility in the soft segments, as evidenced by the shift to lower relaxation temperatures.

Similarly, the peaks at −6.2 °C (TPU/bio-plasticizer 50/10) and −14 °C (TPU/bio-plasticizer 50/5) correspond to the plasticization of the hard segments. The observed decrease in relaxation temperatures suggests that the bio-plasticizer effectively reduces the rigidity of these segments, making their relaxation more detectable within the tested temperature range. Thus, the TSD curves confirm that the bio-plasticizer significantly enhances the molecular mobility of both the soft and hard segments in TPU, demonstrating good compatibility and effective plasticization.

For the TSD analysis of PVC/bio-plasticizer blends, the TSD curves provide valuable insights into the interaction between PVC and the bio-plasticizer, highlighting their compatibility and plasticization efficiency.

As shown in Figure 3, pure PVC (red curve) displays a dominant relaxation peak at 79 °C, attributed to dipolar relaxation and charge detrapping processes within its rigid polymer matrix. This high-temperature relaxation reflects PVC’s restricted molecular mobility and strong intermolecular interactions.

Upon blending with the bio-plasticizer, the PVC/bio-plasticizer system (blue curve) shows a dominant relaxation peak at −0.5 °C. The intermediate position of this peak, between those of the individual components, indicates effective molecular interactions and successful plasticization. The shift to a lower relaxation temperature compared to neat PVC suggests that the bio-plasticizer enhances polymer chain mobility by reducing intermolecular forces.

Furthermore, the presence of a single dominant peak in the blend confirms good compatibility, signifying a well-mixed system without phase separation. This effective interaction leads to homogeneous plasticization, where the bio-plasticizer is well-integrated into the PVC matrix, improving flexibility while maintaining structural integrity.

These findings demonstrate the bio-plasticizer’s potential as a highly compatible and efficient alternative to conventional plasticizers, supporting the development of more sustainable and flexible PVC-based materials.

As shown in Figure 4, the TSD curves of PVC/TPU blends exhibit a single dominant relaxation peak, positioned between the peaks of pure PVC (red curve) and pure TPU (blue curve). These intermediate peaks represent the combined relaxation behavior of the PVC and TPU polymer chains, indicating strong molecular interactions between the two components. The presence of a single major peak, rather than distinct peaks for each polymer, suggests the formation of a well-mixed interphase, reflecting good compatibility and minimizing phase separation.

The PVC/TPU (10/5) blend exhibits a small low-temperature shoulder, suggesting some degree of phase inhomogeneity. In contrast, the ternary PVC/TPU/bio-plasticizer blend displays a single relaxation peak, with no signs of phase segregation, indicating strong intermolecular interactions and a uniform phase morphology, as further supported by the SEM analysis discussed later.

The well-mixed interphase contributes to improved mechanical properties and a more uniform dielectric response, demonstrating that TPU acts as an effective modifier for PVC without inducing significant phase separation. This compatibility arises from the distinct contributions of TPU’s hard and soft segments. The hard segments enhance intermolecular interactions, reinforcing the blend’s mechanical integrity, while the soft segments improve flexibility and toughness by facilitating molecular mobility. This synergistic interaction allows TPU to combine the rigidity and strength of PVC with enhanced elasticity and impact resistance, leading to a well-balanced material with superior performance.

**Figure 5:**
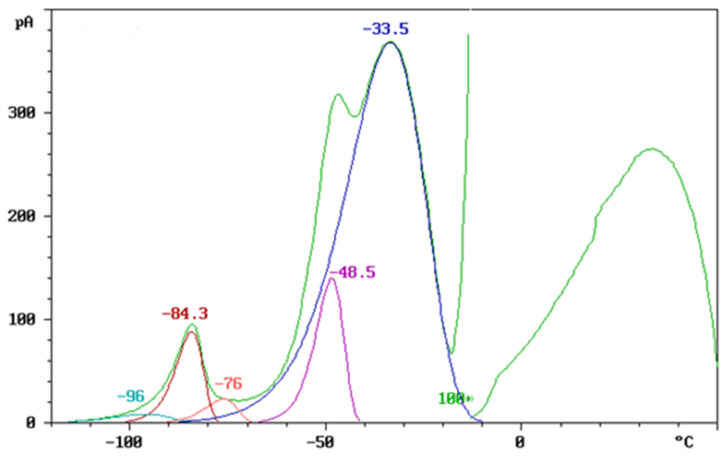
TSD result and resolved peaks for bio-plasticizer, glycerol diacetate monolaurate.

The TSD analysis of the bio-plasticizer, glycerol diacetate monolaurate, was performed to investigate its behavior in polymer blends. The result, as shown in Figure 5, reveals a glass transition temperature ($T_{g}$) at −35 °C, suggesting its potential to enhance flexibility. Additionally, multiple peaks were observed in the TSD curve, indicating that the bio-plasticizer is likely not a pure compound, but contains undisclosed components or contaminants, possibly introduced during its manufacturing process. Furthermore, the observed peaks may result from the movement of different stages of the molecule, which could produce distinct relaxation transitions [34].

#### 3.1.2. TSD Peak Maxima Trends

**Figure 6:**
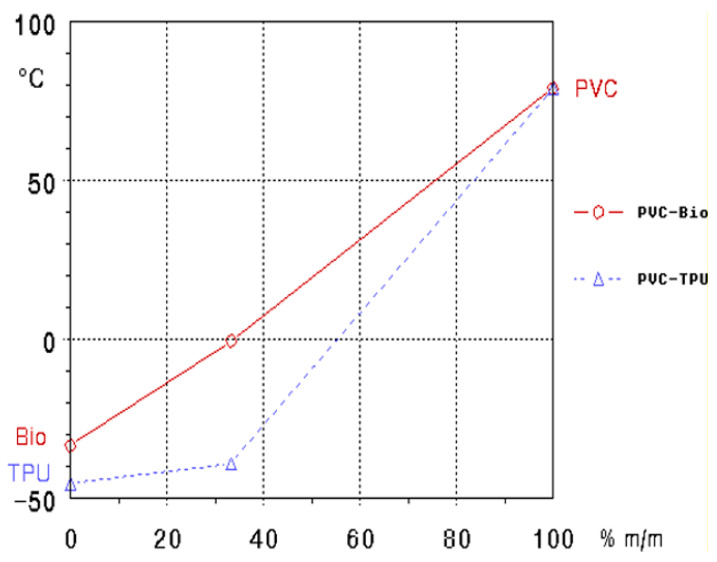
TSD peak maxima of PVC/bio-plasticizer and PVC/TPU blends.

As shown in Figure 6, the behavior of the TSD peak maxima (i.e., the temperature at which maximum depolarization occurs during the heating process) illustrates the effects of the bio-plasticizer and TPU on PVC blends. In the case of the PVC/bio-plasticizer system (red line), a near-linear trend is observed: as the bio-plasticizer content increases, the TSD peak maxima progressively decreases, reflecting the typical plasticizing effect of reducing the glass transition temperature ($T_{g}$). This behavior is attributed to the bio-plasticizer weakening intermolecular interactions, particularly the dipole–dipole forces between PVC chains, leading to enhanced flexibility and a lower $T_{g}$ of the polymer system.

In contrast, the PVC/TPU blends (blue dashed line) display a less linear trend. As the TPU content increases in the PVC blend, there is a more significant reduction in the TSD peak maxima, particularly at higher TPU concentrations. TPU, being a thermoplastic elastomer, introduces greater flexibility to the blend, disrupting the rigid PVC matrix. This suggests TPU’s ability to significantly modify the dipolar relaxation behavior of PVC, lowering its ($T_{g}$). Notably, at 50 phr (33.3%) TPU, the Tg is lowered more than expected, indicating that the TPU used in these experiments acts as a highly efficient plasticizer in terms of Tg reduction. Overall, while increasing the content of either the bio-plasticizer or the TPU resulted in a decrease in the TSD peak maxima of PVC blends, TPU is more effective at lowering the $T_{g}$, making it particularly suitable for applications that demand enhanced flexibility, elasticity, and performance at lower temperatures.

**Figure 7:**
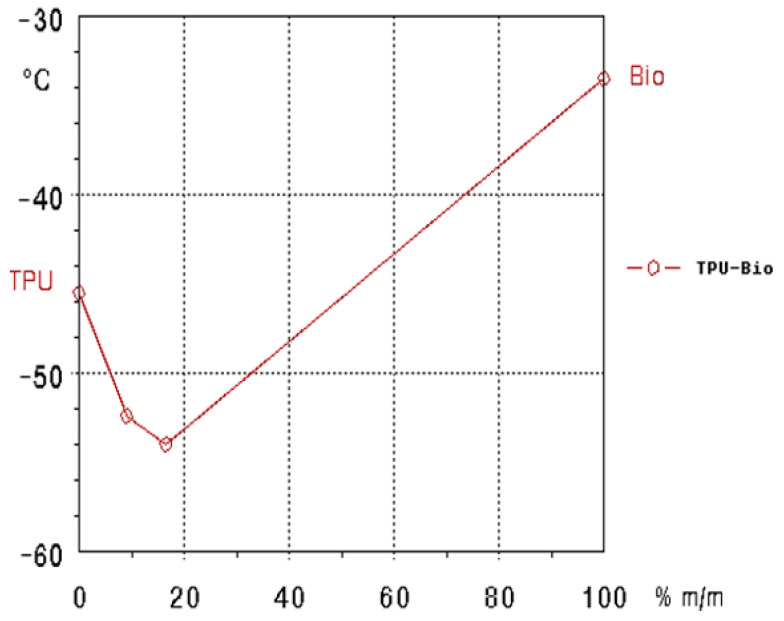
TSD peak maxima of TPU/Bio-plasticizer blend.

The TSD peak maxima of the TPU/bio-plasticizer blend (Figure 7) exhibits an interesting trend with a distinctive minimum. This behavior occurs because the $T_{g}$ of the bio-plasticizer is higher than that of the apolar soft segment of TPU. At lower bio-plasticizer content, it facilitates the mobility of TPU’s soft segments, causing a decrease in the TSD peak maxima. However, as the bio-plasticizer content increases, it begins to dominate the matrix and hinders this segmental mobility, resulting in a steady increase in the TSD peak maxima. This trend demonstrates the dual role of the bio-plasticizer: promoting flexibility at lower concentrations while progressively restricting molecular movement at higher concentrations. The enhanced flexibility at lower temperatures makes this blend particularly well-suited for applications in environments where maintaining flexibility under sub-zero conditions is crucial.

### 3.2. FTIR Analysis of Glycerol Diacetate Monolaurate

**Figure 8:**
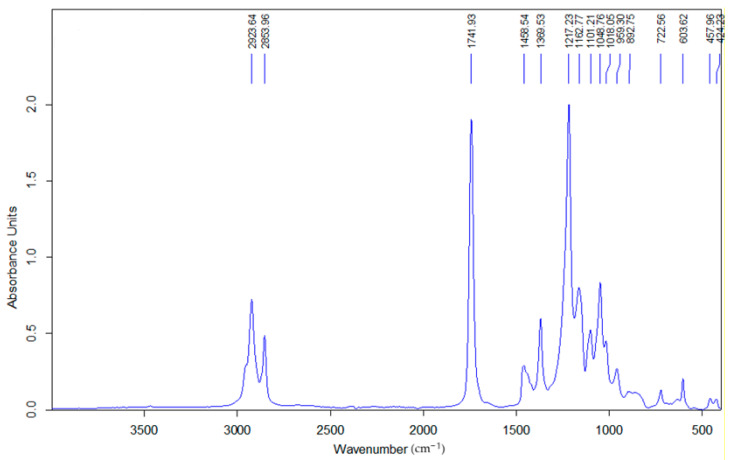
FTIR spectra of glycerol diacetate monolaurate.

The FTIR spectrum of glycerol diacetate monolaurate (Figure 8) confirms its ester-functionalized structure. A strong C=O stretching band at 1741.9 cm^−1^, along with C–O stretching in the 1100–1272 cm^−1^ range, validates the presence of ester bonds. Peaks at 2923.6 cm^−1^ and 2853.9 cm^−1^, corresponding to aliphatic (-CH_2_-) groups, further confirm the laurate moiety and expected molecular structure.

Beyond these characteristic peaks, additional absorption bands suggest trace impurities or residual byproducts from synthesis. The peaks at 722.5 cm^−1^ and 603.6 cm^−1^ indicate the presence of unreacted fatty acid esters or free lauric acid, implying incomplete esterification. Weak absorptions at 1458.5 cm^−1^ and 1369.5 cm^−1^, assigned to C–H bending vibrations, suggest residual mono- or diacylglycerols that were not fully converted. These findings align with the TSD analysis, which revealed molecular heterogeneity, likely due to its sensitivity to minor structural variations. However, the low intensity of these secondary components in FTIR suggests their presence in minimal amounts, with negligible impact on overall material performance.

### 3.3. Dynamic Mechanical Analysis (DMA) of Polymer Blends

**Figure 9:**
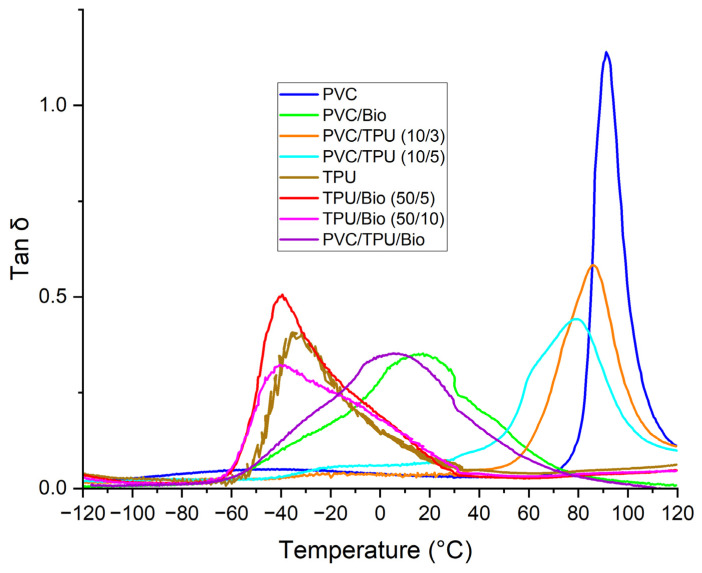
DMA curves of polymer blend samples.

The tan δ curve obtained from DMA measurements (Figure 9) provides key insights into the molecular mobility, phase interactions, and glass transition behavior of the polymer blends. The peak positions and intensities reflect variations in chain flexibility and component compatibility.

Pure PVC exhibits a distinct $T_{g}$ at 91.2 °C, indicating its rigid structure and restricted molecular motion. The incorporation of a bio-plasticizer significantly reduces $T_{g}$, suggesting enhanced flexibility and increased free volume within the polymer matrix. Notably, the PVC/bio-plasticizer (100/50) formulation shows a substantial $T_{g}$ shift to 17.3 °C, confirming strong plasticization effects and good compatibility.

Blending TPU with PVC also lowers $T_{g}$. The PVC/TPU blends (10/3 and 10/5) exhibit $T_{g}$ shifts to 85.6 °C and 78.6 °C, respectively, without distinct secondary transitions from individual components, indicating good phase miscibility. However, the PVC/TPU (10/5) blend displays a minor low-temperature shoulder, suggesting incomplete phase homogeneity, possibly due to TPU’s soft segments forming localized domains.

For pure TPU, $T_{g}$ appears at –38 °C. The addition of a bio-plasticizer further lowers it to −40.6 °C and −42.6 °C for TPU/bio-plasticizer (50/5) and (50/10), respectively, confirming its role in enhancing molecular mobility. Notably, the ternary PVC/TPU/bio-plasticizer blend exhibits a single $T_{g}$ at 5.5 °C, with no indication of phase separation, suggesting strong intermolecular interactions and a well-integrated phase structure.

Overall, these results highlight the influence of TPU and bio-plasticizers on the thermal and molecular behavior of polymer blends, ultimately shaping their mechanical performance and suitability for various applications.

### 3.4. Scanning Electron Microscopy (SEM) Analysis of Polymer Blends 

**Figure 10:**
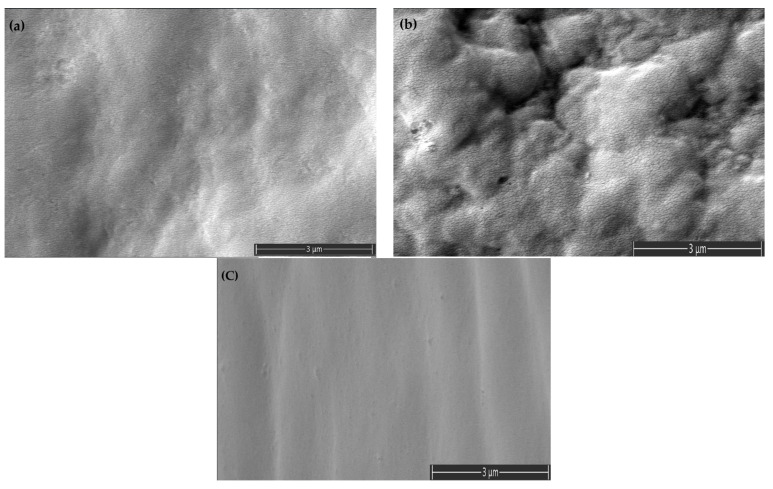
SEM micrographs of (**a**) PVC/TPU (10/3), (**b**) PVC/TPU (10/5), and (**c**) PVC/TPU/bio-plasticizer (10/2/5).

The SEM micrographs (Figure 10) illustrate the morphological characteristics of PVC/TPU (10/3), PVC/TPU (10/5), and PVC/TPU/bio-plasticizer blends, highlighting the effects of composition on phase distribution and miscibility.

In Figure 10a, the PVC/TPU (10/3) blend exhibits a relatively smooth and uniform morphology, suggesting good TPU dispersion within the PVC matrix and favorable phase interactions. However, increasing TPU content in the PVC/TPU (10/5) (Figure 10b) results in greater surface roughness with visible microphase domains, indicating partial phase separation. This suggests that, at higher TPU concentrations, localized TPU-rich regions form due to limited compatibility.

In contrast, the PVC/TPU/bio-plasticizer blend (Figure 10c) displays a more homogeneous and integrated morphology, with reduced phase separation and enhanced interfacial adhesion. The bio-plasticizer likely facilitates better TPU dispersion by increasing polymer chain mobility and reducing interfacial tension between the phases.

These morphological observations align with DMA and TSD results, which confirm variations in molecular interactions and phase behavior across the different formulations.

### 3.5. Results of Mechanical Measurements

**Figure 11:**
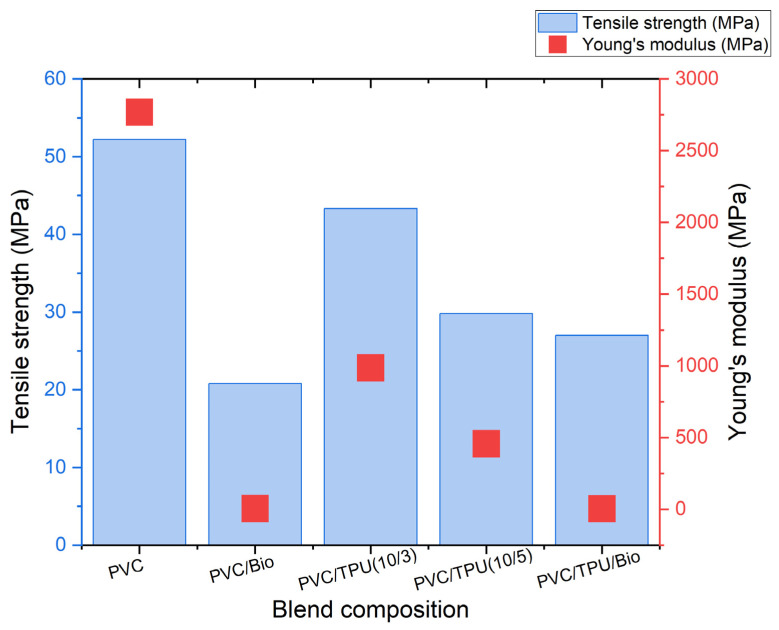
Tensile strength and Young’s modulus of polymer blends.

**Figure 12:**
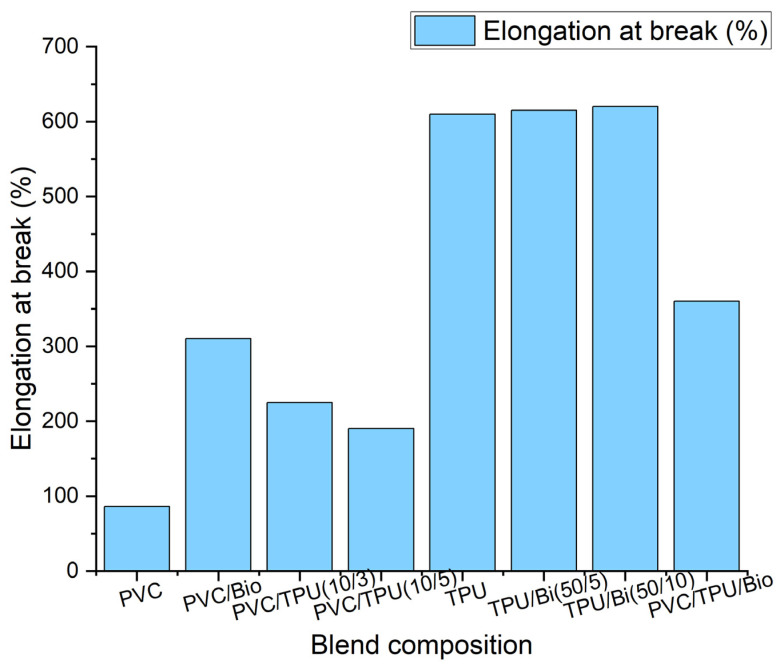
Elongation at the break of polymer blends.

**Figure 13:**
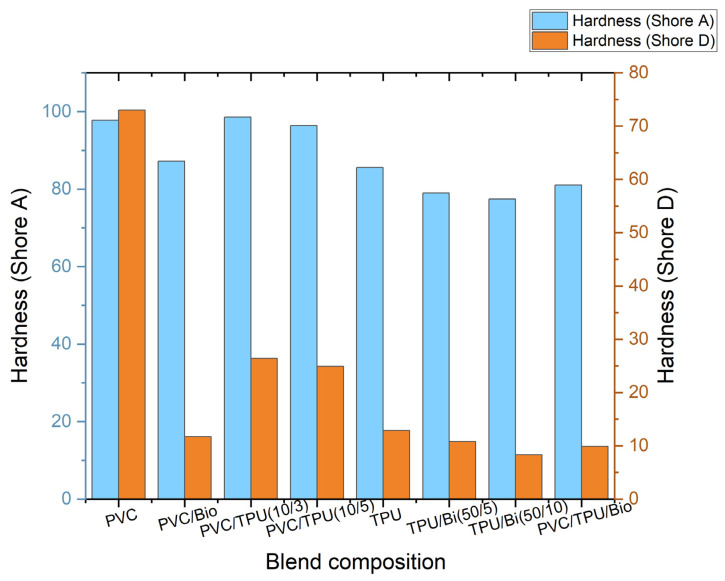
Hardness of polymer blends.

The wide range of mechanical properties of the polymer blends produced using the three components, namely PVC, TPU, and bio-plasticizer, are presented in Table 2 and Figure 11, Figure 12 and Figure 13.

#### 3.5.1. Tensile Properties

The tensile properties, including tensile strength, elongation at break, and Young’s modulus of polymer blends as a function of composition, are summarized in Figure 11 and Figure 12.

As shown in Figure 11 and Figure 12, pure PVC exhibited the highest tensile strength among the PVC-based samples (52.2 MPa), with a high Young’s modulus (2768 MPa) and limited elongation at break (86%), reflecting its intrinsic rigidity. TSD (Figure 3) and DMA (Figure 9) further support this observation, as pure PVC displays a single, sharp relaxation peak, indicating restricted chain mobility.

Incorporating 50 phr of bio-plasticizer into PVC significantly reduced tensile strength (20.8 MPa) but greatly enhanced elasticity, as evidenced by a sharp decrease in Young’s modulus (5.9 MPa) and a notable increase in elongation at break (310%). These findings confirm effective plasticization, increasing chain mobility and flexibility. While the reduced tensile strength limits its use in load-bearing applications, the enhanced ductility makes PVC/bio-plasticizer blends suitable for flexible products such as medical tubing and packaging films.

Blending PVC with TPU improved flexibility and elongation at break while retaining higher tensile strength than the bio-plasticized PVC. The PVC/TPU (10/3) blend exhibited a tensile strength of 43.3 MPa, a Young’s modulus of 987.2 MPa, and an elongation at break of 225%. These improvements can be attributed to good phase compatibility between PVC and TPU, as confirmed by TSD and DMA, which show a single dominant relaxation peak (Figure 4 and Figure 9, respectively). Additionally, SEM images reveal a more homogeneous morphology (Figure 10a), indicating effective TPU dispersion within the PVC matrix. The improved compatibility allows TPU to impart toughness and flexibility while PVC maintains overall structural stability. The combination of moderate tensile strength, enhanced flexibility, and good toughness makes PVC/TPU (10/3) suitable for protective films, impact-resistant coatings, and semi-flexible industrial hoses.

In contrast, the PVC/TPU (10/5) blend exhibited a minor low-temperature shoulder in both TSD and DMA curves and increased surface roughness with microphase domains in SEM (Figure 10b), indicating partial phase separation. This reduced phase homogeneity hindered stress transfer, leading to a further decrease in tensile strength (27.8 MPa), Young’s modulus (457.5 MPa), and elongation at break (194%). Despite the decrease in mechanical efficiency, the improved flexibility and moderate strength make PVC/TPU (10/5) a potential candidate for soft-touch grips, flexible tubing, and vibration-damping components, where higher elasticity is required without complete loss of structural integrity.

Pure TPU exhibited high flexibility, with a tensile strength of 52.9 MPa, a low Young’s modulus (8.2 MPa), and exceptional elongation at break (610%), characteristic of its segmented block copolymer structure, where hard segments provide mechanical strength and soft segments confer elasticity. Incorporating a bio-plasticizer into TPU progressively reduced tensile strength while enhancing elongation. TPU/bio-plasticizer (50/5) retained relatively high tensile strength (49.9 MPa) with increased elongation (615%). However, at 10 phr of bio-plasticizer, tensile strength decreased further (34.2 MPa), while elongation peaked (620%), highlighting the trade-off between flexibility and mechanical strength due to increased molecular mobility.

Notably, incorporating TPU into the PVC/bio-plasticizer (100/50) blend (PVC/TPU/bio-plasticizer at 100/20/50) resulted in a well-balanced mechanical performance. This blend exhibited improved elasticity (Young’s modulus of 4.9 MPa) and elongation at break (360%) compared to PVC/bio-plasticizer and PVC/TPU blends, without a significant reduction in tensile strength (27 MPa). The strong intermolecular interactions, as evidenced by TSD and DMA results, along with the homogeneous morphology observed in SEM (Figure 10c), indicate enhanced compatibility, contributing to superior mechanical properties. The optimized balance of strength, elasticity, and elongation makes this blend ideal for applications requiring bending or movement, such as flexible cables, soft-touch automotive components, and dynamic structural elements.

#### 3.5.2. Results of Hardness Testing

Figure 13 illustrates the effect of blend composition on the hardness of polymer blends, as measured using Shore A and Shore D scales.

PVC exhibited the highest hardness value due to its rigid molecular structure and strong intermolecular forces. The incorporation of a bio-plasticizer significantly reduced hardness, as it disrupts the rigid PVC structure, enhancing flexibility while reducing indentation resistance. This trend aligns with its lower $T_{g}$ observed in the DMA results, confirming increased molecular mobility. Similarly, in TPU/bio-plasticizer blends, an increase in bio-plasticizer content resulted in a decrease in hardness, as the bio-plasticizer softens the TPU matrix, improving flexibility while decreasing resistance to deformation.

For PVC/TPU blends, increasing TPU content resulted in a noticeable decrease in hardness. As a thermoplastic elastomer, TPU introduces elasticity and reduces stiffness, leading to lower hardness values compared to pure PVC. Notably, the PVC/TPU (10/3) and (10/5) blends maintain relatively high hardness values, indicating that TPU imparts flexibility while preserving structural integrity. This behavior is supported by DMA and TSD findings, where a well-defined relaxation transition indicates good phase compatibility, preventing excessive softening.

The ternary blend, which combines PVC, TPU, and bio-plasticizer, shows moderate hardness, reflecting a balanced composition that offers both flexibility and toughness. This makes it ideal for applications where an intermediate hardness is preferred for optimal comfort and performance.

Overall, the mechanical properties described above demonstrate that blends of PVC, TPU, and bio-plasticizer can exhibit a broad range of properties, offering versatile applications that cannot be achieved by any of the individual components alone.

### 3.6. Thermal Degradation Behavior

**Figure 14:**
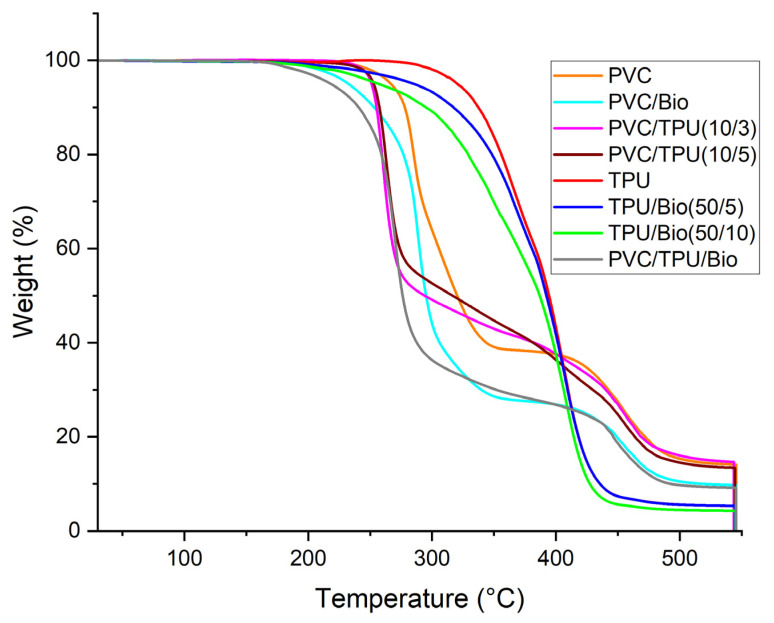
TGA curves of polymer blend samples.

The TGA curves (weight loss vs. temperature) in Figure 14 provide insights into the thermal stability and degradation mechanisms of pure polymers and their modified formulations.

Pure PVC undergoes a two-step degradation process. The first major weight loss occurs at 270 °C due to dehydrochlorination, leading to HCl release and polyene formation. The second degradation stage, associated with the breakdown of the conjugated backbone, occurs around 425 °C. The final degradation step at 481 °C results in a relatively high char residue (14%), attributed to PVC’s chlorine content, which enhances flame retardancy but also affects its overall thermal stability.

TPU, in contrast, exhibits the highest thermal stability among the tested samples, with a single major degradation step. Thermal decomposition initiates at 315 °C due to urethane bond cleavage, followed by the breakdown of soft and hard segments, with rapid weight loss around 390 °C, leading to nearly complete volatilization by 450 °C. The minimal char residue (5%) suggests TPU undergoes near-total decomposition without significant residue formation.

The incorporation of a bio-plasticizer into PVC reduces its thermal stability. Its initial degradation temperature decreases to 261 °C, indicating that the plasticizer promotes earlier decomposition due to increased molecular mobility and volatility. The final degradation step shifts slightly to 473 °C, with a reduced char residue (10%). A similar trend is observed in TPU/bio blends. TPU/bio (50:5) degrades at 302 °C, with its main degradation step at 390 °C, whereas TPU/bio (50:10) begins decomposing at 290 °C, with the primary degradation at 380 °C. The lower char residue (4–5%) confirms that higher bio-plasticizer content exacerbates thermal instability, consistent with the increased molecular mobility observed in DMA results.

PVC/TPU (10/3) and PVC/TPU (10/5) blends exhibit slightly lower onset degradation temperatures (256 °C and 254 °C, respectively) compared to pure PVC (270 °C). This can be attributed to the catalytic effect of HCl release from PVC, which may accelerate the breakdown of TPU’s urethane bonds. However, the second degradation stage remains relatively high (440–445 °C), indicating that while TPU does not significantly enhance PVC’s initial thermal resistance, it influences its decomposition behavior. Notably, TPU moderates the sharp degradation steps of PVC, resulting in a more gradual degradation profile, which can be advantageous for applications requiring controlled thermal stability.

The PVC/TPU/bio-plasticizer blend shows a further reduced onset degradation temperature (247 °C) due to the plasticizer’s lower thermal stability. However, its main degradation temperature remains similar to PVC/TPU blends, suggesting that TPU contributes to structural integrity despite the presence of the plasticizer. The final char residue (9%) is lower than that of PVC/TPU blends but higher than TPU/bio blends, reflecting a balance between degradation resistance and flexibility. This formulation provides an optimal trade-off between processability and thermal performance, making it suitable for applications where flexibility is prioritized over maximum thermal resistance.

## 4. Conclusions

This study demonstrates that PVC, TPU, and bio-plasticizer blends are both thermodynamically and technologically compatible, making them promising materials for industrial applications. TSD and DMA revealed molecular interactions and compatibility in PVC/bio-plasticizer, PVC/TPU, TPU/bio-plasticizer, and PVC/TPU/bio-plasticizer blends. The PVC/TPU blends, particularly the PVC/TPU (100/30) formulation, exhibited better phase compatibility than PVC/TPU (100/50), as evidenced by smooth morphology in scanning electron microscopy (SEM) and a single relaxation peak in both DMA and TSD. In TPU/bio-plasticizer blends, shifts in TSD peak temperatures for soft and hard segment relaxations demonstrated enhanced chain mobility due to the bio-plasticizer. Nonlinear TSD peak maxima trends in PVC/TPU blends highlighted TPU’s ability to disrupt the rigid PVC matrix, acting as an efficient modifier of dipolar relaxation. TGA analysis revealed that while the bio-plasticizer reduces thermal stability, TPU improves the degradation profile when incorporated into PVC.

The wide range of enhanced mechanical and thermal properties exhibited by these blends confirms their technological compatibility. Blending PVC, TPU, and bio-plasticizer synergistically combines their strengths: PVC’s rigidity and strength, TPU’s elasticity, and the bio-plasticizer’s flexibility-enhancing properties. For instance, PVC/bio-plasticizer blends improve elongation at break and flexibility while maintaining structural integrity, making them suitable for low-stress applications. TPU/bio-plasticizer blends show enhanced elasticity with slightly reduced tensile strength, making them ideal for highly elastic materials. The ternary PVC/TPU/bio-plasticizer blend offers an optimized balance of properties, including increased elasticity and elongation at break with minimal compromise in tensile strength, as well as moderate hardness, making it suitable for applications requiring both flexibility and durability.

In conclusion, this study confirms that the combination of PVC, TPU, and bio-plasticizer produces compatible polymer blends that effectively bridge the performance gaps between the individual components. The verified thermodynamic and technological compatibility of these blends opens opportunities for tailoring properties at different mixing ratios, enabling optimization for diverse applications.

## Figures and Tables

**Table 1:** Composition of tested blends (parts per hundred, phr).

	PVC	PVC/Bio	PVC/TPU (10/3)	PVC/TPU (10/5)	TPU	TPU/Bio(50/5)	TPU/Bio(50/10)	PVC/TPU/Bio
PVC base mixture	100	100	100	100	-	-	-	100
TPU	-	-	30	50	100	100	100	20
Bio plasticizer	-	30	-	-	-	10	20	50
CaZn stab.	1.2	1.2	1.2	1.2	-	-	-	1.2
E-wax	0.3	0.3	0.3	0.3	-	-	-	0.3

**Table 2:** Mechanical properties of tested blends.

	PVC	PVC/Bio	PVC/TPU (10/3)	PVC/TPU (10/5)	TPU	TPU/Bio(50/5)	TPU/Bio(50/10)	PVC/TPU/Bio
Tensile strength (MPa)	52.2	20.8	43.3	27.8	52.9	49.9	34.2	27
Young’s modulus (MPa)	2768	5.9	987.2	457.5	8.2	6.9	3.5	4.9
Elongation at break (%)	86	310	225	194	610	615	620	360
Hardness (Shore A)	97.78	87.2	98.6	99.4	85.6	79	77.4	81
Hardness (Shore D)	83	11.7	26.4	24.9	12.9	10.8	8.3	9.9

## Data Availability

The research data are available upon request to the corresponding author.
